# Quantitative evaluation of lesion response heterogeneity for superior prognostication of clinical outcome

**DOI:** 10.1007/s00259-024-06764-0

**Published:** 2024-05-31

**Authors:** Ojaswita Lokre, Timothy G. Perk, Amy J. Weisman, Rajkumar Munian Govindan, Song Chen, Meijie Chen, Jens Eickhoff, Glenn Liu, Robert Jeraj

**Affiliations:** 1 AIQ Solutions, 8000 Excelsior Dr Suite 400, Madison, WI 53717 United States of America; 2 Department of Nuclear Medicine, The First Hospital of China Medical University, Shenyang, Liaoning China; 3 Department of Medical Physics, University of Wisconsin–Madison, Madison, WI United States of America

**Keywords:** Tumor heterogeneity, Lung cancer, Lymphoma, FDG PET/CT, Computational methods, Clinical imaging, Prognostication of clinical outcome

## Abstract

**Purpose:**

Standardized reporting of treatment response in oncology patients has traditionally relied on methods like RECIST, PERCIST and Deauville score. These endpoints assess only a few lesions, potentially overlooking the response heterogeneity of all disease. This study hypothesizes that comprehensive spatial-temporal evaluation of *all* individual lesions is necessary for superior prognostication of clinical outcome.

**Methods:**

[^18^F]FDG PET/CT scans from 241 patients (127 diffuse large B-cell lymphoma (DLBCL) and 114 non-small cell lung cancer (NSCLC)) were retrospectively obtained at baseline and either during chemotherapy or post-chemoradiotherapy. An automated TRAQinform IQ software (AIQ Solutions) analyzed the images, performing quantification of change in regions of interest suspicious of cancer (lesion-ROI). Multivariable Cox proportional hazards (CoxPH) models were trained to predict overall survival (OS) with varied sets of quantitative features and lesion-ROI, compared by bootstrapping with C-index and t-tests. The best-fit model was compared to automated versions of previously established methods like RECIST, PERCIST and Deauville score.

**Results:**

Multivariable CoxPH models demonstrated superior prognostic power when trained with features quantifying response heterogeneity in *all* individual lesion-ROI in DLBCL (C-index = 0.84, *p* < 0.001) and NSCLC (C-index = 0.71, *p* < 0.001). Prognostic power significantly deteriorated (*p* < 0.001) when using subsets of lesion-ROI (C-index = 0.78 and 0.67 for DLBCL and NSCLC, respectively) or excluding response heterogeneity (C-index = 0.67 and 0.70). RECIST, PERCIST, and Deauville score could not significantly associate with OS (C-index < 0.65 and *p* > 0.1), performing significantly worse than the multivariable models (*p* < 0.001).

**Conclusions:**

Quantitative evaluation of response heterogeneity of *all* individual lesions is necessary for the superior prognostication of clinical outcome.

**Supplementary Information:**

The online version contains supplementary material available at 10.1007/s00259-024-06764-0.

## Introduction

Heterogeneity of lesion response to treatment has been observed in many cancers including metastatic melanoma [1], lung [2], colorectal [3], lymphoma [4] and prostate cancers [5]. In cases where a heterogeneous response is present, it is currently difficult for clinicians to assess whether a patient is benefiting from their current treatment regimen and to decide how to proceed with treating the patient. When the overall disease burden may be improving, but there is still a significant number of lesions not responding to therapy, systemic treatment intensification may be required. On the other hand, in cases where the majority of lesions are favorably responding to treatment, targeting individual progressing lesions using localized ablation may extend benefit and prolong survival [6, 7]. Therefore, a better understanding of treatment response heterogeneity at the lesion level of analysis has significant implications for clinical decision making and strong potential for improving patient outcomes, reducing costs, and increasing drug development efficiency in clinical trials.

Due to the high prevalence of intra-patient response heterogeneity across many types of metastatic cancer [8], measurement of treatment response in all individual disease sites across the body is imperative. Positron emission tomography (PET) and computed tomography (CT) are commonly used to monitor response to therapy in metastatic cancer patients, as they provide a non-invasive method of measuring anatomic and functional changes across the whole body. Measuring response in all disease sites, particularly with whole body PET/CT images, is labor-intensive. Thus, a limited subset of lesions selected based on size or PET uptake is used for the sake of feasibility [9, 10]. As a result, treatment response heterogeneity is often difficult to appreciate as the treating provider only has access to semi-quantitative or descriptive reports interpreted from a subset of lesions present.

Several guidelines to standardize treatment response reporting, particularly in context of clinical trials, where improvements in the “mean” has been used to determine which treatment option was better, have been developed. These include the Response Evaluation Criteria In Solid Tumors (RECIST) [9], World Health Organization (WHO) [11] Criteria, Positron Emission tomography Response Criteria in Solid Tumors (PERCIST) [10] and Deauville Score [12]. The RECIST and WHO criteria were initially developed in the era of cytotoxic chemotherapy and therefore used changes in tumor size as an endpoint for clinical trials, assuming that a higher proportion of tumor shrinkage would reflect improvement in overall survival. RECIST and WHO guidelines stated that “mixed response” was uncommon, thus the assessment of a limited number of lesions was adequate to prioritize agents and choose dosing regimens for late-phase clinical trials. Based on that, RECIST 1.1 [9] later changed the number of assessed lesions from 10 to 5, and preserved the unidimensional measurement (WHO used 2 dimensional measurement) as a way to simplify yet provide “sufficient” standardization for clinical trials. PERCIST was developed for [^18^F]Fluoro-2-deoxy-2-D-glucose (FDG) PET with a similar goal of standardizing response and allowing comparison between trials. PERCIST is predominantly based on change in the lesions with the highest FDG uptake. Limitations include change in non-target and new lesions, which defers to clinical judgement or a subjective determination of whether it meets “unequivocal” progression. Nevertheless, all these criteria rely on the “sum of change” in target lesions, thus ignoring the impact of each individual lesion in the evaluation of treatment response heterogeneity.

Multiple studies have shown strong associations between FDG standardized uptake value (SUV) metrics and survival [13–15], but results across these studies are inconsistent. A study by Kurtipek et al. [16]. showed that average lesion uptake (SUV_mean_) and metabolic tumor volume (MTV) have a significant association with survival time, but maximum lesion uptake (SUV_max_) showed no statistical significance. On the other hand, other studies showed SUV_max_ to have a strong prognostic value for survival [17–19]. Additionally, prognostic significance of measurement of tumor heterogeneity [20–22] has shown potential, but its exact impact on patient outcomes has not been determined. One of the main limitations was that for patients with multiple lesions, manual assessment is impractical and has poor reproducibility. Thus, automation is necessary to assess all lesions to improve ability and performance.

In this study, we explored a methodology aimed at providing clinicians with advanced automated TRAQinform IQ (AIQ Solutions, Madison, WI) software analysis. This analysis comprehensively characterizes lesion-level regions of interest (lesion-ROI), enabling early detection of both anatomical and functional changes and assessment of treatment response heterogeneity. Additionally, it evaluates how these changes impact the prognostic value of FDG PET/CT scans. This is the first study that looks at the impact of variation in lesion-ROI and heterogeneity on the prognostication of outcomes. Two cohorts of subjects with non-small cell lung cancer and diffuse large B-cell lymphoma were used in the study. Statistical modelling was used to simulate how a clinician would use quantitative features to prognosticate overall survival of patients. We hypothesized that quantitative evaluation of response heterogeneity of *all* individual lesions is necessary for superior prognostication of clinical outcome.

## Methods

### Patient population

Patients with metastatic non-small cell lung cancer (NSCLC) and diffuse large B-cell lymphoma (DLBCL), who received repeat FDG PET/CT imaging, were selected for our analysis. Selection of these cancer types, where FDG PET/CT is commonly used as a standard-of-care treatment response assessment tool [23–29], allowed for comparison of the applied methodology across different tumor types.

The DLBCL dataset from the randomized phase III CALGB50303 trial, which studied the efficacy of rituximab in combination with two different chemotherapy regimens, included 127 patients with multiple scans available for analysis [30, 31]. The NSCLC dataset from the multi-center ACRIN 6668 trial studying the use of early FDG PET/CT to predict long-term clinical outcome (survival) after definitive chemoradiotherapy included 114 patients with multiple scans available for analysis [13, 32]. The dataset included all data with at least the first two time points, which were the baseline and follow-up scans. Full information for each dataset, including publications of the primary objectives where relevant, is shown in Table 1.

**Table 1:** Information of clinical trials and imaging data that was included for retrospective analysis in this study. Clinical benefit is presented for all patients who received baseline images. The proportion of patients with heterogenous change (having both decreasing or disappeared and increasing or new lesion-ROI) was calculated for all patients who received baseline and follow-up images

Dataset	CALGB50303 - DLBCL	ACRIN 6668 - NSCLC
Clinical Trial Number	NCT00118209	NCT00083083
Imaging Timepoints	Baseline 2–3 weeks after cycle 2 of chemotherapy	Baseline 14–16 weeks post radiotherapy
Disease	Diffuse large B-cell lymphoma	Non-small cell lung cancer
Treatments	Dose-adjusted etoposide, prednisone, vincristine, cyclophosphamide, doxorubicin, and rituximab (DA-EPOCH-R) with standard rituximab, cyclophosphamide, doxorubicin, vincristine, and prednisone (R-CHOP)	Chemoradiotherapy
Patients who received baseline and follow-up FDG PET/CT images *(N)*	127	114
Number of lesion-ROI on baseline *Median [range]*	9 [0-110]	3 [0–15]
Overall survival *(Days)* *Median [range]*	1845 [82–3293]	753 [118–2326]
Percentage of patients with heterogeneous change *(%)*	51/127 (40.1%)	101/114 (88.6%)

Both studies had rigorous quality control of the FDG PET/CT imaging with centralized standardization based on phantoms as specified in their respective imaging protocols [33, 34]. PET/CT images were reviewed by an experienced research associate or technologist regarding technical specifications such as dosage, timing, acquisition, and reconstruction, and checked if they were compliant with the protocol. Images that were not compliant were either rectified or removed.

For the DLBCL patients, images were acquired at baseline and 2–3 weeks after cycle 2 of chemotherapy. For NSCLC, images were baseline and 12–16 weeks post radiation therapy (and at least 4 weeks post chemotherapy). Patients were required to fast for 4 h and have blood glucose levels less than 200 mg/dL before the FDG injection. The FDG dose was not mandated; the recommended dose was 0.14 to 0.21 MBq/kg (approximately 10 to 20 MBq). PET/CT scanning took place 50 to 70 min after FDG injection and included the body from mid cervical spine to proximal femurs. Reconstruction of PET images was performed in accordance with the imaging protocols for both studies [33, 34]. Scanner information is reported in Table 2.

**Table 2:** Scanner manufacturers

	DLBCL (*N* = 254)			NSCLC (*N* = 228)	
	Siemens (*N* = 150)	GE (*N* = 67)	Philips (*N* = 37)	Siemens (*N* = 111)	GE (*N* = 117)
Patient Sex, n Male/Female/Unknown	88/62/0	41/26/0	28/9/0	71/33/7	75/42/0
Patient Age, years Median [range]	57 [20–82]	59 [20–77]	55 [23–79]	61 [1–90]	68 [36–83]
Scanner Model	1094 (*n* = 31) 1080 (*n* = 29) Biograph 64 (*n* = 22) Biograph 40 (*n* = 29) 1023 (*n* = 20) Biograph 128 (*n* = 9) Biograph 20 (*n* = 7) Biograph 6 (*n* = 3)	Discovery ST (*n* = 58) Discovery LS (*n* = 3) Discovery STE (*n* = 3) Discovery 690 (*n* = 3)	GEMINI TF TOF 16 (*n* = 33) Gemini TF(C) (*n* = 1) Gemini TF (*n* = 1) GEMINI TF TOF 64 (*n* = 1) Ingenuity TF PET/CT (*n* = 1)	1023 (*n* = 40) 1080 (*n* = 34) 1024 (*n* = 17) 1094 (*n* = 17) 1062 (*n* = 2) 1093 (*n* = 1)	Discovery ST (*n* = 61) Discovery LS (*n* = 33) Discovery STE (*n* = 16) Discovery RX (*n* = 7)
Slice thickness, mm	2.50 (*n* = 55) 3.00 (*n* = 30) 1.50 (*n* = 25) 2.00 (*n* = 13) 4.00 (*n* = 10) 5.00 (*n* = 8) 3.40 (*n* = 6) 2.62 (*n* = 1) 3.19 (*n* = 1) 3.07(*n* = 1)	3.27 (*n* = 61) 4.25 (*n* = 5) 2.50 (*n* = 1)	5.00 (*n* = 31) 2.50 (*n* = 2) 3.00 (*n* = 2) 2.00 (*n* = 1) 4.00 (*n* = 1)	2.50 (*n* = 58) 3.40 (*n* = 16) 2.00 (*n* = 14) 3.00 (*n* = 12) 2.40 (*n* = 5) 4.00 (*n* = 5) 5.00 (*n* = 1)	3.27 (*n* = 84) 4.25 (*n* = 33)

### Lesion-ROI level augmentative software analysis

For this study, analysis was performed by TRAQinform IQ software (AIQ Solutions, Madison, WI).The TRAQinform IQ software performs quantitative analysis of heterogeneity of change in volume and tracer-uptake using automated matching [35, 36] of lesion-ROI between FDG PET/CT images. TRAQinform IQ software also performs automatic organ segmentation, trained using the method described in Weisman et al., to provide locations of lesion-ROI [37]. The organ segmentation model is trained to include all malignancies within the segmented organ. Maximum intensity projections (MIPs) of the organ segmentation output of all patients were manually reviewed to ensure no major failures occurred. TRAQinform IQ is a software-only medical device intended for use by trained medical professionals.

From every PET/CT image, TRAQinform IQ software extracted single time-point image features from both baseline and follow-up scans in each individual lesion-ROI: SUV_max_ (the highest SUV within lesion-ROI), SUV_mean_ (the average SUV in lesion-ROI), Volume (the total volume of lesion-ROI), SUV_total_ (the total SUV in lesion-ROI), SUV_hetero_ (standard deviations of all uptake in lesion-ROI), SUV_peak_ (defined as the average value in a 1 cm^3^ sphere centered around the highest uptake voxel in the lesion-ROI) and lesion-ROI count (the number of identified lesion-ROI). Additionally, response features, defined as the change in each feature, were calculated. TRAQinform IQ software tracked each individual lesion-ROI between the baseline and follow-up scans, and then it categorized as new, increasing, stable, decreasing, or disappeared based on the ± 30% change in SUV_total_ approximating the repeatability coefficients of repeat FDG PET/CT scans [38].

This categorization allowed for the extraction of additional image heterogeneity features that quantify the heterogeneity of lesion-ROI changes which included: the count of lesion-ROI in each category (e.g., the count of new lesion-ROI), the fraction of lesion-ROI in each category (e.g., the fraction of lesion-ROI classified as new), and specific characteristics (such as SUV_max_, SUV_mean_, SUV_hetero_, SUV_total_, and Volume) of lesion-ROI in each category (e.g., the highest SUV value of increasing lesion-ROI). Supplementary Table 1 contains a definition list of all the extracted features.

### Comparator response criteria

Three standard methods for patient-level treatment response evaluation were implemented as comparators: RECIST [9], PERCIST [10] and Deauville score [39] for the DLBCL cohort and RECIST and PERCIST for the NSCLC cohort. The standard-of-care (SOC) treatment response assessment evaluations were automated using the outputs from TRAQinform IQ’s individual lesion-ROI assessment.

In the automated RECIST evaluation, CT measurements were obtained from each of the PET identified lesion-ROI, and the RECIST target lesions were selected based on the 5 largest volumes of all identified lesion-ROI (no more than 2 per auto segmented organ). The largest long-axis diameter (LAD) was selected by measuring the LAD across every axial slice of the selected lesion-ROI.

In the automated PERCIST evaluation, SUV_peak_ of the lesion-ROI with the highest SUV_max_ in each of the scans was used to select 1 target lesion.

In the automated Deauville score evaluation, the lesion-ROI were classified into five categories based on SUV_peak_ compared to the SUV_mean_ of aorta or the SUV_mean_ of the liver. Score 1–no uptake, Score 2- SUV_peak_ less than or equal to aorta SUV_mean_, Score 3- SUV_peak_ more than aorta SUV_mean_ but less than or equal to liver SUV_mean,_ Score 4- SUV_peak_ higher than the liver SUV_mean_ but no higher than 3 times the liver SUV_mean_, and Score 5– SUV_peak_ more the 3 times the liver SUV_mean_.

A subset of 20 patients (10 DLBCL and 10 NSCLC) were manually assessed by two nuclear medicine physicians (SC − 13 years of experience, MC − 11 years of experience) to verify the automated treatment response evaluation for RECIST, PERCIST, and Deauville score.

### Treatment outcome prediction

This study implemented multivariable Cox proportional hazards (CoxPH) regression models to predict overall survival (OS) utilizing features extracted from the TRAQinform IQ software produced lesion-ROI specific reports (Supplementary Table 1).

To mitigate the risk of overfitting, a feature selection process preceded the integration of features into the models. Initially, univariable p-values for all features were computed, and features with a univariable p-value below a threshold of 0.2 were considered for inclusion in the model, the threshold previously used to successfully correlate features with survival [40]. Subsequently, a bootstrapped stepwise backward selection model using the Bayesian Information Criterion (BIC) was applied [41, 42]. This approach systematically identified parsimonious features by selecting the most relevant ones across multiple bootstrap samples. From these iterations, percentages were generated, representing the frequency of feature selection across the samples. To arrive at a final set of features for integration into the model, only those features ranking within the top 40th percentile were chosen. This brought the number of features down to fewer than 14 features per model. Two analyses were performed to evaluate the need for prognostics models of OS account for all lesion-ROI rather than subgroups and for response of each lesion-ROI rather than whole patient trends.

### Lesion-ROI subgroup analysis

First, to assess the value of using all lesion-ROI for analyzing a subject, multivariable CoxPH models were trained, including all features, for different number of lesion-ROI as input:

- All lesion-ROI: all lesion-ROI were assessed.
- 5 biggest lesion-ROI: the five largest lesion-ROI by volume were assessed, with a limit of two lesion-ROI per organ, as prescribed by RECIST criteria.
- 1 hottest lesion-ROI: the single lesion-ROI with the highest SUV_peak_ value was assessed, as prescribed by PERCIST criteria.

### Feature subgroup analysis

Second, to assess the need to include change of all lesion-ROI, multivariable CoxPH models were trained with different feature sets, extracted for all lesion-ROI, given as input. (Supplementary Table 1)

- All features: Baseline (BL: single timepoint whole patient features on the baseline scans), Follow-up (FU: single timepoint whole patient features on the follow-up scans), Patient-level Response (Response: change in each single-timepoint whole patient feature from baseline to follow-up), and intra-patient heterogeneity features.
- Baseline + Follow-up + Patient-level Response (BL + FU + Response): Baseline and Follow-up along with percent change in each single-timepoint whole patient feature from baseline to follow-up.
- Follow-up (FU): Only single timepoint whole patient features on the follow-up scans.
- Baseline (BL): Only single timepoint whole patient features on the baseline scans.

### Comparison to previously existing methods

The top-performing model from our previous analyses was compared against previously established methods.

RECIST, PERCIST, and Deauville score each output distinct response categories for every patient. These were standardized into a 1–5 numerical scale reflecting best to worst prognosis assigned by each criterion. For RECIST/PERCIST the scale was 1: complete response (CR/CMR), 2: partial response (PR/PMR), 3: stable disease (SD/ SMD), 4: progressive disease (PD/PMD). For Deauville the scale was 1: Score 1, 2: Score 2, 3: Score 3, 4: Score 4 and 5: Score 5.

Univariable CoxPH models were fit using RECIST, PERCIST or Deauville score as features for comparison with the selected model. Moreover, the selected model also underwent comparisons with models fitted using individual predictive features previously identified as predictive in these trial cohorts, post-treatment SUV_peak_ and pre-treatment molecular tumor volume (Volume) for NSCLC [13, 43, 44], and change of SUV_max_ for DLBCL [45, 46].Univariable CoxPH models were fit using RECIST, PERCIST or Deauville Score as features for comparison with the selected model. Moreover, the selected model also underwent comparisons with models fitted using individual predictive features previously identified as predictive in these trial cohorts, post-treatment SUV_peak_ and pre-treatment molecular tumor volume (Volume) for NSCLC [13, 43, 44], and percent change of SUV_max_ for DLBCL [45, 46].

### Statistical analysis

Overall survival (OS) was defined as time from the baseline FDG PET/CT scan to the date of patient death. OS for surviving patients was censored at the date of the last survival assessment. Censoring was used for patients that did not die during the monitoring period of the trials [44, 46].

The performance of survival predictions of the CoxPH models was assessed using the concordance index (c-index), which is a generalization of the area under the receiver operating characteristic curve that accounts for the prediction of time to event such as overall survival with censored observations [47]. A c-index of 1 indicates perfect model performance, and indicates the model was able to order a set of patients correctly according to their risk.

The stability of each model was ensured by employing a robust bootstrapping method where the original data was resampled to generate 1000 unique bootstrap samples [48, 49]. For each combination of input feature and lesion-ROI, a CoxPH model was then trained on every bootstrap sample, producing a C-index for each sample outcome. The final C-index of the specific input combination was calculated as the median C-index across the 1000 bootstrap model outcomes. Comparisons of C-indices between models were conducted using a paired t-test based on the bootstrap estimated standard errors of paired differences.

The hazard ratio (HR), its 95% confidence interval (CI), and associated p-value were also derived for the final model.

The Proportional Hazard assumptions for the CoxPH models were verified prior to analysis by performing Scaled Schoenfeld residuals statistical testing [50]. Statistical analyses were carried out using R (version 4.3.2).

**Fig. 1:**
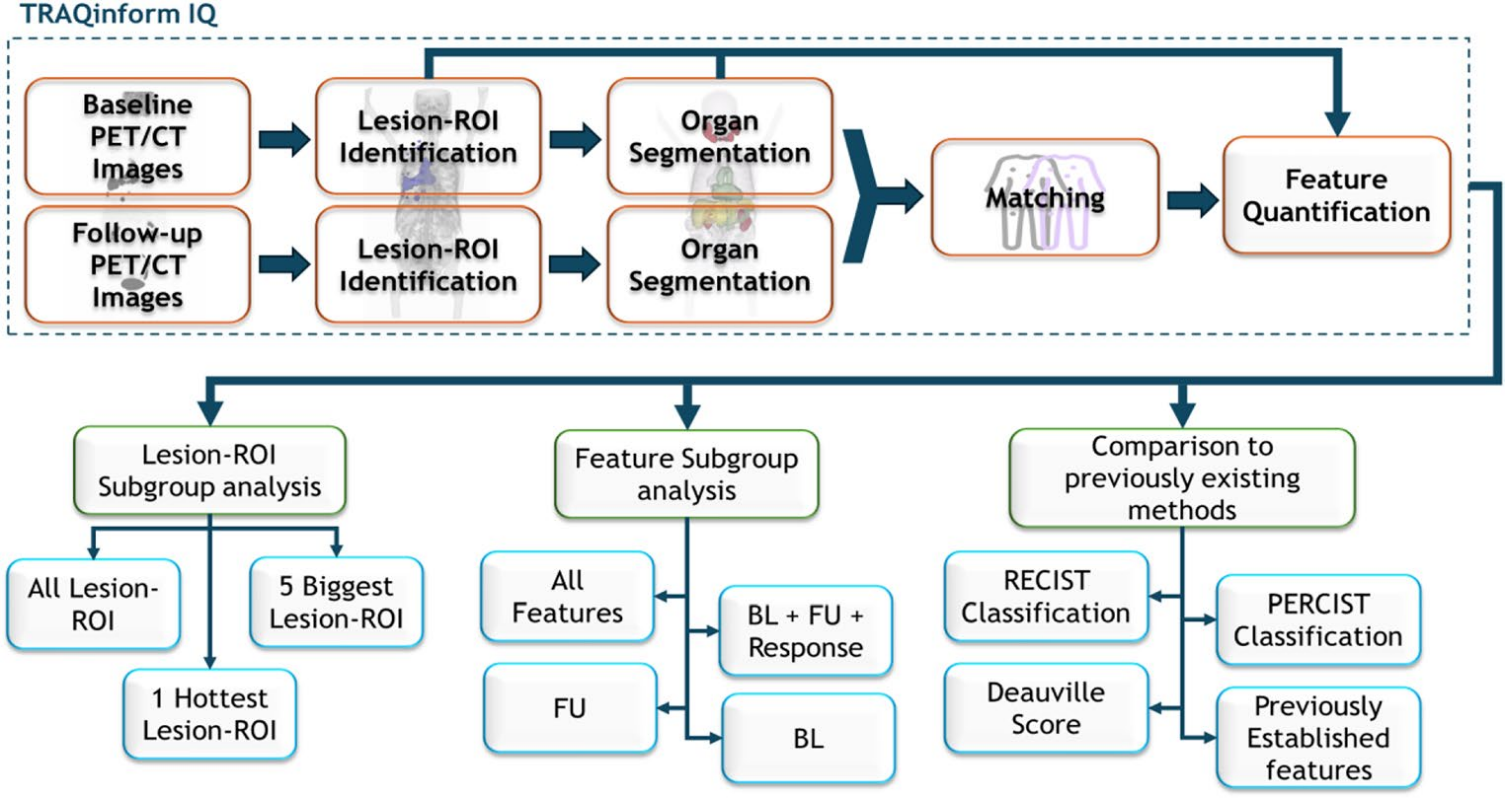
Summary scheme of all the steps involved in the methodology of the study

A summary scheme of the methodological steps described in the study has been included in Fig. 1.

## Results

### Patient and dataset information

**Fig. 2:**
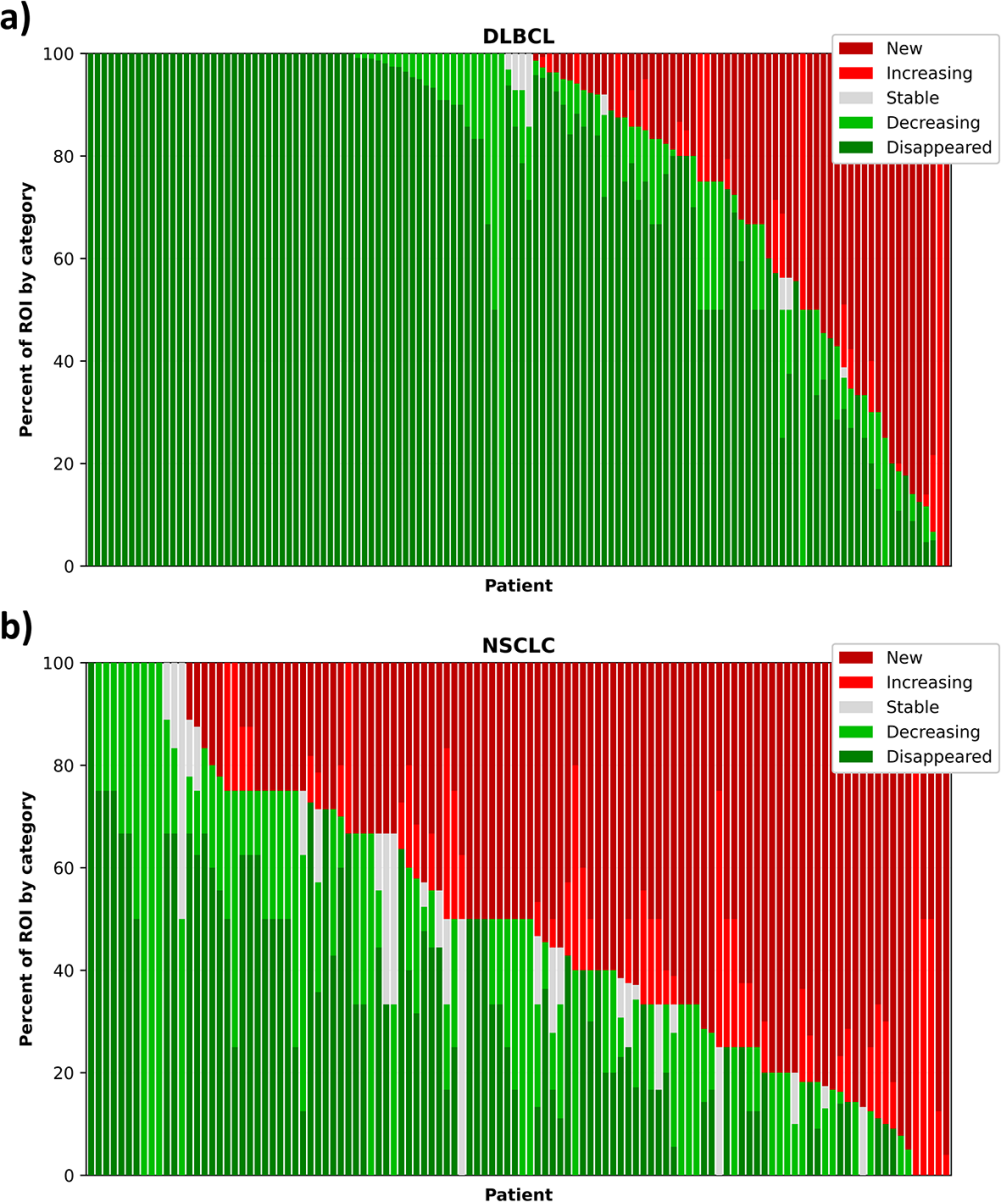
Heterogeneity in the **a** DLBCL and **b** NSCLC datasets. Each bar represents a patient, and the height of each color represents the proportion of lesion-ROI in that patient in each response category

**Fig. 3:**
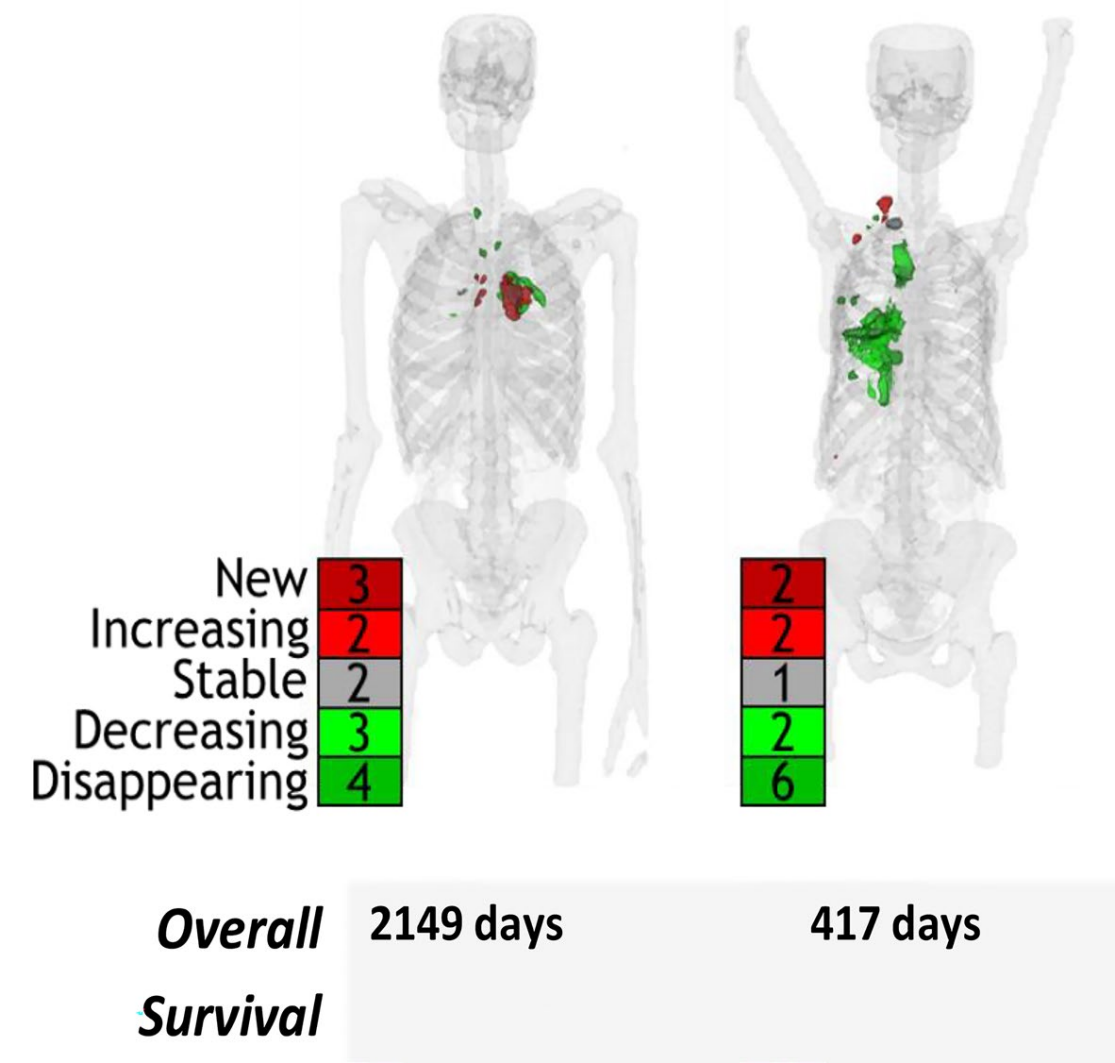
Example patients with heterogeneous response

A total of 241 patients were included in the analysis across the two datasets. A summary of the number of lesion-ROI, duration of benefit, and percent of patients with heterogenous response, defined as having at least one new/increasing and one disappeared/decreasing lesion-ROI at follow up, of each study is shown in Table 1. The NSCLC patients had fewer lesion-ROI per patient (median of 3 lesion-ROI) and shorter overall survival (median of 753 days OS) compared to the DLBCL patients (median of 9 lesion-ROI, median of 1845 days OS). Heterogeneous response was identified in 63% (152/241) patients. The heterogeneity of lesion-ROI change is depicted in Fig. 2. Example patients with heterogeneous response are shown in Fig. 3.

### Performance of automated comparator response criteria

When comparing automated implementation of the standard of care guidelines, the automated methods agreed with the manual assessment in most cases (17/20, 16/20, 10/10, for RECIST, PERCIST, and Deauville, respectively). In all NSCLC patients, the automated methods agreed perfectly. Differences were noted in DLBCL patients when different target lesions were selected, likely due to sub-optimal selection when human observers select the target lesions.

### Performance of survival models

The results of all the different models have been summarized in Table 3.

**Table 3:** Performance of CoxPH models trained with 1000 bootstrap samples. Inputs were varied based on number of lesion-ROI and different feature combinations. The best model was compared to previously established predictors. C-index (*±* standard deviation) was obtained. P-values of the overall model were obtained using Score (logrank) test. Models included all information unless specified in the table

Comparison	Input model	DLBCL		NSCLC	
		C-index	P-value	C-index	P-value
Lesion-ROI subgroup	All features, all lesion-ROI	**0.84 ± 0.04**	< 0.001	**0.71 ± 0.03**	< 0.001
	5 biggest lesion-ROI	0.78 ± 0.06	< 0.001	0.67 ± 0.03	0.006
	1 hottest lesion-ROI	0.77 ± 0.05	< 0.001	0.68 ± 0.03	< 0.001
Feature subgroup	All features, all lesion-ROI	**0.84 ± 0.04**	< 0.001	**0.71 ± 0.03**	< 0.001
	BL + FU + Patient-level Response	0.67 ± 0.06	0.03	0.70 ± 0.03	< 0.001
	FU	0.64 ± 0.08	0.2	0.65 ± 0.03	< 0.001
	BL	0.67 ± 0.06	0.03	0.64 ± 0.03	< 0.001
Previously Established Predictors	All features, all lesion-ROI	**0.84 ± 0.04**	< 0.001	**0.71 ± 0.03**	< 0.001
	RECIST	0.59 ± 0.05	0.14	0.50 ± 0.01	0.47
	PERCIST	0.58 ± 0.05	0.2	0.51 ± 0.01	0.51
	Deauville	0.62 ± 0.07	0.25	-	-
	Global SUV_peak_2	-	-	0.57 ± 0.03	0.01
	Global Volume 1	-	-	0.62 ± 0.03	< 0.001
	Percent Change Global SUV_max_	0.58 ± 0.06	0.3	-	-

**Fig. 4:**
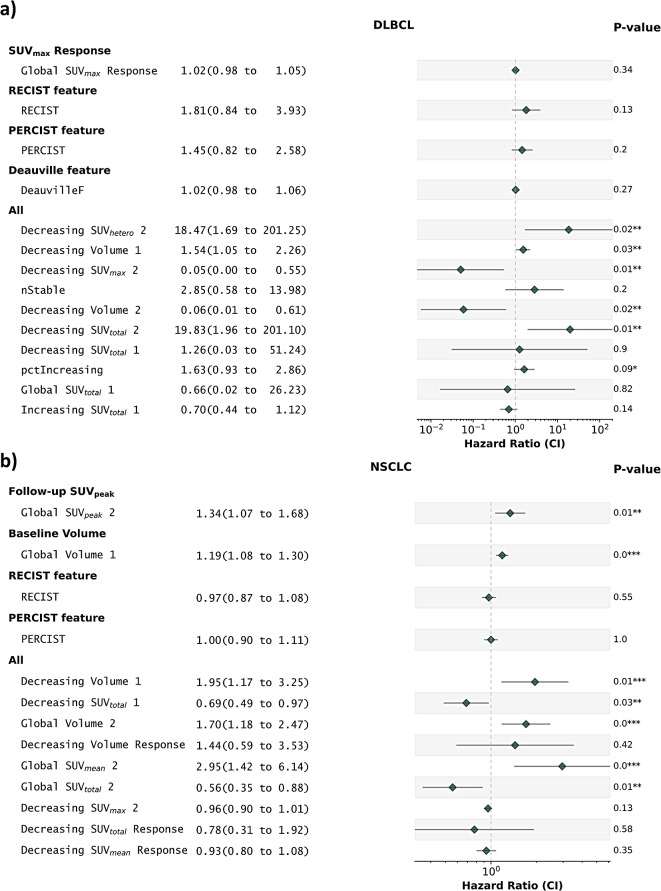
Forest plots for **a**) DLBCL and **b**) NSCLC depicting the Hazard ratios (95% CI) and corresponding p-values comparing the best model from our analysis to the previously established predictors

The analysis revealed that the highest performance in both datasets was achieved when all features and all lesion-ROI were included for training with a C-index of 0.84 (*p* < 0.001) for DLBCL and 0.71 (*p* < 0.001) for NSCLC. The features selected for each of these models is shown in Fig. 4.

### Lesion-ROI subgroup analysis

**Fig. 5:**
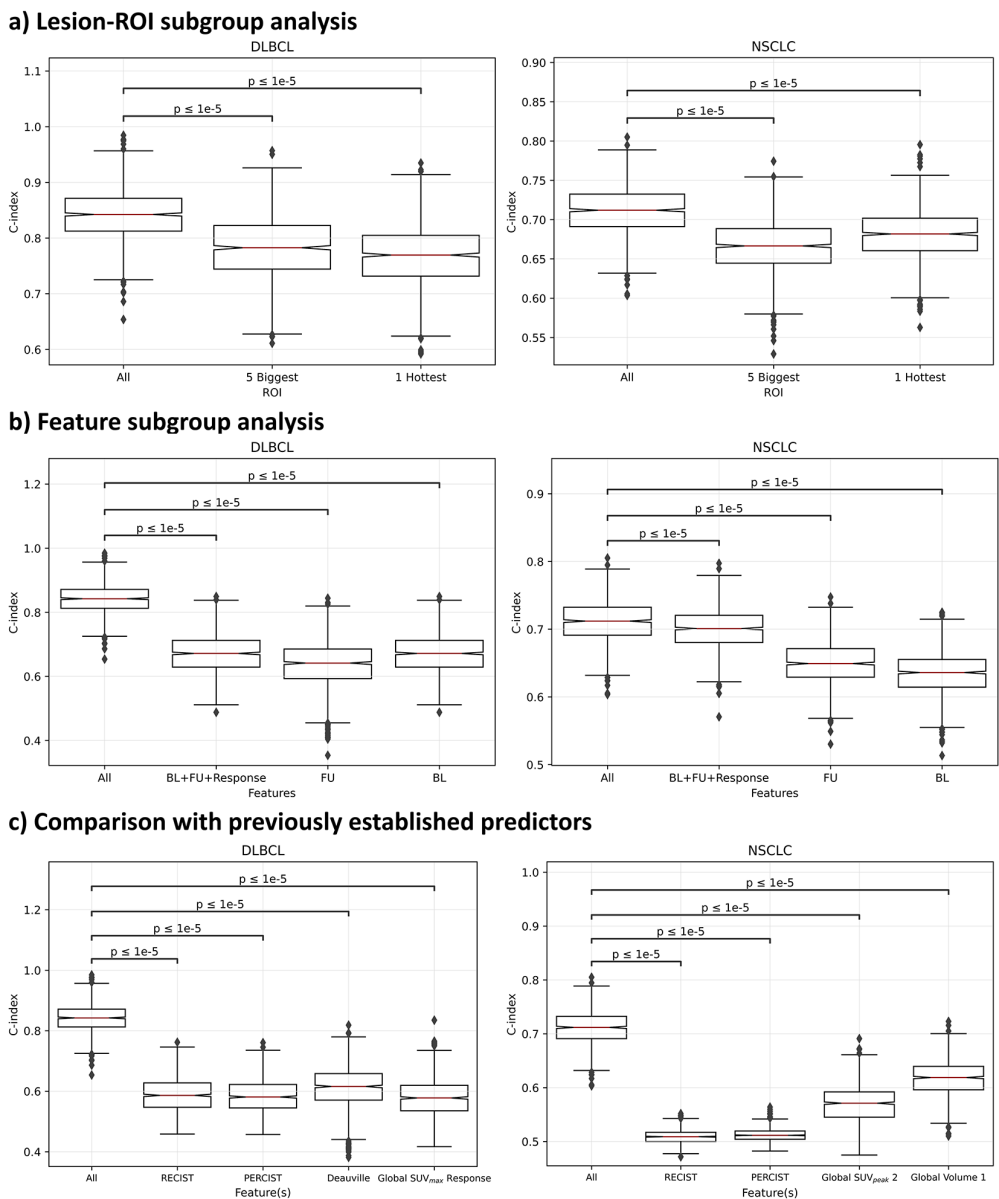
Box plots depicting the c-index from the coxPH models trained on 1000 bootstrap samples **a**) Lesion-ROI Subgroup Analysis **b**) Feature Subgroup Analysis **c**) Comparison with previously established predictors. The p-values are calculated using a paired t-tests with Bonferroni correction

When CoxPH models only had information for the lesion-ROI with the highest SUV_peak_ there was still a significant prognostic power (C-index = 0.77, *p* < 0.001 for DLBCL and C-index = 0.68, *p* < 0.001 for NSCLC). This was also true when the model had information on up to 5 biggest lesion-ROI (C-index = 0.78, *p* < 0.001 for DLBLC and C-index = 0.67, *p* = 0.006 for NSCLC). Paired t-tests identified that these models had statistically significantly lower predictive power compared the models trained with information from all lesion-ROI (p-values < 0.0001) as shown in Fig. 5 (a).

### Feature subgroup analysis

If CoxPH models only had information of all lesion-ROI at baseline (BL), there still would be significant prognostic power (C-index = 0.67, *p* = 0.03 for DLBCL and C-index = 0.64, *p* < 0.001 for NSCLC). However, models with only information at the time of follow-up imaging (FU) were only prognostic for NSCLC (C-index = 0.64, *p* = 0.2 for DLBCL and C-index = 0.65, *p* = 0.006 for NSCLC). Models had significant prognostic power when patient-level baseline information was combined with patient-level follow-up and whole patient response (BL + FU + Response, C-index = 0.67, *p* = 0.03 for DLBCL and C-index = 0.70, *p* < 0.001 for NSCLC). However, the performance of each of these models was significantly lower than the highest-performing model (*p* < 0.0001) as shown in Fig. 5 (b).

### Comparison to previously existing methods

RECIST, PERCIST, and Deauville score were not significant predictors of OS (C-index < 0.65 and *p* > 0.1), with significantly worse outcome predictions than the multivariate models (*p* < 0.001). Notably, the previously applied predictive variable in DLBCL in percent change in SUV_max_ was not a significant predictor of OS (C-index = 0.58, *p* = 0.3), which was significantly worse than the multivariate model (*p* < 0.001). In NSCLC, SUV_peak_ on the follow-up scan (C-index = 0.57, *p* = 0.01) and volume on the baseline scan (C-index = 0.62, *p* < 0.001) were both significant predictors of OS, but this was significantly lower than the multivariate model (*p* < 0.001), as shown in Fig. 5 (c). The hazard ratios (95% CI) and their corresponding p-values for the above models are displayed in Fig. 4.

## Discussion

In this work, we hypothesized that quantitative evaluation of response heterogeneity of *all* individual lesions is necessary for superior prognostication of clinical outcome. To evaluate this, TRAQinform IQ software (AIQ Solutions) was used for automated quantification and analysis of multiple FDG PET/CT images to aid in comprehensively characterizing full-body lesion-ROI-wise early anatomical and functional change in metastatic non-small cell lung cancer (NSCLC) and diffuse large B-cell lymphoma (DLBCL) patients. The aim was to implement multivariable Cox proportional hazards models trained to predict overall survival, simulating how a physician would use information to manage patients based on the information provided to them. Bootstrapping was implemented to understand the stability and variability of models and to allow for statistical comparison of models.

The first comparison was using quantitative features extracted from varied groups of lesion-ROI: all lesion-ROI, the five biggest lesion-ROI based on volume but no more than two per organ, and the single hottest-ROI based on SUV_peak_. While all the models were significant predictors of overall survival in both cancers, models trained with all lesion-ROI included had significantly superior performance than those trained with features extracted from only a few lesion-ROI, establishing that all information from all ROI is necessary for superior prognostication of outcomes. This difference was larger in DLBCL (0.77 to 0.84) than in NSCLC (0.67 to 0.71), likely due to the larger numbers of lesion-ROI in DLBLC than in NSCLC (median 9 vs. 3). Likely this impact would be even larger in patient populations with higher disease burden. The five biggest lesion-ROI and the single hottest lesion-ROI were tested as they are the selection criteria for target lesions in RECIST and PERCIST. Future work will include identification of characteristics of lesion-ROI that provide the most meaningful information to prognosticate outcomes.

Next, models were compared when training with only baseline single-timepoint SUV metrics, only follow-up single-timepoint SUV metrics, combining baseline, follow-up and response of SUV metrics, and the addition of lesion-ROI-level heterogeneity metrics. Statistically significantly superior results at predicting OS were observed when heterogeneity of response of lesion-ROI was included in the models. This emphasizes the importance of understanding response heterogeneity when prognosticating outcomes.

The automated standard of care RECIST and PERCIST criteria, implemented on both datasets, were inadequate predictors based on survival outcomes. This is likely because they only assess a limited number of lesions, new lesions, or high uptake lesions, but fail to include critical information on response or heterogeneity in either uptake or change, which we found to be critical for predicting outcomes. Similarly in the Deauville criteria, the prognostic power was likely poor because the scoring system effectively only assesses a single, most metabolically active lesion-ROI at the second timepoint, which does not account for change during treatment nor intra-patient change heterogeneity.

This analysis stressed the limits of standard of care assessment as they are constrained to small numbers of lesions since it is impractical for manual assessment of all possible disease on every image and quantifying the change over the course of treatment [35]. On the other hand, TRAQinfrom IQ software quantifies this change for all lesion-ROI and allows for more complex analyses to better predict clinical outcomes of patients from their imaging.

The main finding of this work is not the ability of models to prognosticate outcomes, but rather that models had superior performance when factoring in response of all lesions rather than subsets of lesions. The results suggest that when treating patients with multiple lesions, physicians would have a better understanding of the prognosis of patients if quantitative information of response of all lesions was available to them. This could allow clinicians to make better decisions on how to treat patients with metastatic cancer.

Previous analyses of the ACRIN 6668 trial (source of the NSCLC data) identified post-treatment SUV_peak_ and pre-treatment molecular tumor volume (MTV) as a significant predictor of OS [13, 43, 44]. Our analysis supported these measures as significant univariate predictors. Previous analysis of the CALGB50303 trial (source of the DLBCL data) showed that percent change of SUV_max_ was a predictor of OS [45, 46]. In our analyses, this was not a significant predictor of survival. This difference could be due to differences in the assessment tools and that only a subset of the total patients was made available for analysis by TRAQinform IQ.

Several noteworthy features selected by the multivariable all-feature models were heterogeneity features focusing on the change of individual lesion-ROI, such as decreasing SUV_total_ (SUV_total_ of the decreasing lesion-ROI), decreasing volume (volume of the decreasing lesion-ROI), decreasing SUV_max_ (SUV_max_ of decreasing lesion-ROI), among others. Conversely, the majority of univariable predictors demonstrated minimal to negligible significance in terms of hazard ratios. This underscores the need to use multiple features in tandem to prognosticate patient outcomes.

While this work trained statistical models that could be used to predict outcomes of patients, this was not the aim of this study. These models were trained and evaluated with bootstrapping for the purpose of evaluating if stable and strong predictive models could be trained based on varied sets of information to determine which information created the highest performing model. Validation of models with an external dataset should be performed before using these models for decision making.

The retrospective nature of this study may raise concerns about the applicability of our findings to prospective settings. Given that the data is derived solely from independent singular studies, the transferability of our results to wider populations is potentially limited. Variations in the quality of acquisition, potential biases introduced by cohorts not representative of real-world populations, and changes in imaging technology over time further contribute to the limitations of this study.

To strengthen the robustness and generalizability of our findings, it is crucial to undertake further validation using additional patient datasets encompassing diverse populations. This validation process would serve to mitigate the limitations posed by the retrospective design and the exclusive reliance on singular study data. Additionally, future endeavors should include validation in prospective studies, incorporating different tracers to provide a more comprehensive understanding of the findings. Note that PET quantification can be dependent on scanner capabilities and reconstruction parameters. As the images acquired in this study were part of two prospective clinical trials with strict imaging protocols, further work is needed to ensure these prognostic trends remain on standard of care images across multiple imaging centers.

Furthermore, it is important to acknowledge the limitations of Cox proportional hazards regression and the statistical feature selection in effectively capturing non-linearities within the data. Machine learning models are emerging as alternatives showing superior performance to Cox regression [51, 52]. It is reasonable to expect that superior performance for outcome modeling will also apply when response of all lesions is used. Therefore, future work should investigate the development and implementation of state-of-the-art machine learning models using larger datasets to allow for training and external validation of these models. These advanced models will be instrumental in compensating for the complexities associated with non-linear relationships within the datasets and enhancing the accuracy and reliability of predictive models.

## Conclusion

In this work, we investigated the impact of using TRAQinform IQ software to quantify response heterogeneity of all lesion-ROI on statistical models predicting overall survival patients receiving serial FDG PET/CT images. The best performance was observed when imaging features characterizing the response heterogeneity between scans of *all* lesion-ROI were considered. The models were able to prognosticate outcomes in two different patient populations with varying disease burden and varying incidence of heterogeneity, while standard of care response criteria (RECIST, PERCIST and Deauville score) were not. Use of automated methods like TRAQinform IQ software is necessary to provide the clinician with a more complex analysis of individual patients which allows for better understanding of patient status e.g. treatment response from their imaging exams as the input. The characterization of the treatment response may allow for earlier identification of patients who will fail and to what extent on specific drugs, enabling a more patient-specific approach to optimal treatment decision making.

## Electronic supplementary material

Below is the link to the electronic supplementary material.

Supplementary Material 1

## Data Availability

The datasets were obtained from The Cancer Imaging Archive (TCIA). The Diffuse Large B-Cell lymphoma data is from the dataset NCT00118209 from the NCTN/NCORP Data Archive of the National Cancer Institute’s (NCI’s) National Clinical Trials Network (NCTN). Data was originally collected from clinical trial NCT number NCT00118209, CALGB-50303. The non-small cell lung cancer data is from the clinical trial NCT number NCT00083083, ACRIN6668. All analyses and conclusions in this manuscript are the sole responsibility of the authors and do not necessarily reflect the opinions or views of the clinical trial investigators, the NCTN, the NCORP or the NCI.
