# Supplementary material for: Quantitative evaluation of lesion response heterogeneity for superior prognostication of clinical outcome
Source: Eur J Nucl Med Mol Imaging. 2024 May 31;51(12):3505–17. doi: 10.1007/s00259-024-06764-0 (PMC11445285; doi:10.1007/s00259-024-06764-0)
Supplement: Supplementary file 1 — Supplementary Material 1 [file 259_2024_6764_MOESM1_ESM.docx]

**Supplementary Table 1:** FDG PET/CT image quantification features by feature set.

| Feature | Description | Feature set | | | |
| --- | --- | --- | --- | --- | --- |
|  |  | Baseline (BL) | Follow-up (FU) | Baseline, Follow-up, Patient-level Response (BL + FU + Response) | All ( BL + FU + Response + intra-patient heterogeneity) |
| Global SUV_max_ 1 | SUV_max_ on the baseline scan. | x |  | x | x |
| Global SUV_mean_ 1 | SUV_mean_ on the baseline scan. | x |  | x | x |
| Global SUV_total_ 1 | SUV_total_ on the baseline scan. | x |  | x | x |
| Global Volume 1 | Volume on the baseline scan. | x |  | x | x |
| Global SUV_hetero_ 1 | Mean Standard deviation on the baseline scan. | x |  | x | x |
| Global Count 1 | The number of lesion-ROI on the baseline scan. | x |  | x | x |
| Global SUV_max_ 2 | SUV_max_ on the follow-up scan. |  | x | x | x |
| Global SUV_mean_ 2 | SUV_mean_ on the follow-up scan. |  | x | x | x |
| Global SUV_total_ 2 | SUV_total_ on the follow-up scan. |  | x | x | x |
| Global Volume 2 | Volume on the follow-up scan. |  | x | x | x |
| Global SUV_hetero_ 2 | Mean Standard deviation on the follow-up scan. |  | x | x | x |
| Global Count 2 | The number of lesion-ROI on the follow-up scan. |  | x | x | x |
| Global SUV_max_ Response | Change in SUV_max_ from baseline to follow-up. |  |  | x | x |
| Global SUV_mean_ Response | Change in SUV_mean_ from baseline to follow-up. |  |  | x | x |
| Global SUV_total_ Response | Change in SUV_total_ from baseline to follow-up. |  |  | x | x |
| Global Volume Response | Change in Volume from baseline to follow-up. |  |  | x | x |
| Change SUV_hetero_ response | Change in Mean Standard deviation from baseline to follow-up. |  |  | x | x |
| nDisappeared | The number of disappeared lesion-ROI at follow-up. |  |  |  | x |
| nDecreasing | The number of decreasing lesion-ROI at follow-up. |  |  |  | x |
| nStable | The number of stable lesion-ROI at follow-up. |  |  |  | x |
| nIncreasing | The number of increasing lesion-ROI at follow-up. |  |  |  | x |
| nNew | The number of new lesion-ROI at follow-up. |  |  |  | x |
| pctDisappeared | The percentage of disappeared lesion-ROI at follow-up. |  |  |  | x |
| pctDecreasing | The percentage of decreasing lesion-ROI at follow-up. |  |  |  | x |
| pctStable | The percentage of stable lesion-ROI at follow-up. |  |  |  | x |
| pctIncreasing | The percentage of increasing lesion-ROI at follow-up. |  |  |  | x |
| pctNew | The percentage of new lesion-ROI at follow-up. |  |  |  | x |
| pctIncreasingNew | The percentage of increasing and new lesion-ROI. |  |  |  | x |
| pctDecreasingDissapeared | The percentage of decreasing and disappeared lesion-ROI. |  |  |  | x |
| Increasing SUV_max_ 1 | SUV_max_ at baseline of lesion-ROI that increase. |  |  |  | x |
| Increasing SUV_mean_ 1 | SUV_mean_at baseline of lesion-ROI that increase. |  |  |  | x |
| Increasing SUV_total_ 1 | SUV_total_ at baseline of lesion-ROI that increase. |  |  |  | x |
| Increasing Volume 1 | Volume at baseline of lesion-ROI that increase. |  |  |  | x |
| Increasing SUV_hetero_ 1 | Mean standard deviation at baseline of lesion-ROI that increase. |  |  |  | x |
| Increasing Count 1 | The number of lesion-ROI at baseline that increase. |  |  |  | x |
| Increasing SUV_max_ 2 | SUV_max_ at follow-up of lesion-ROI that increase. |  |  |  | x |
| Increasing SUV_mean_ 2 | SUV_mean_ at follow-up of lesion-ROI that increase. |  |  |  | x |
| Increasing SUV_total_ 2 | SUV_total_ at follow-up of lesion-ROI that increase. |  |  |  | x |
| Increasing Volume 2 | Volume at follow-up of lesion-ROI that increase. |  |  |  | x |
| Increasing SUV_hetero_ 2 | Mean standard deviation at follow-up of lesion-ROI that increase. |  |  |  | x |
| Increasing Count 2 | The number of lesion-ROI at follow-up that increase. |  |  |  | x |
| Increasing SUV_max_ Response | Change in SUV_max_ from baseline to follow-up of lesion-ROI that increase. |  |  |  | x |
| Increasing SUV_mean_ Response | Change in SUV_mean_ from baseline to follow-up of lesion-ROI that increase. |  |  |  | x |
| Increasing SUV_total_ Response | Change in SUV_total_ from baseline to follow-up of lesion-ROI that increase. |  |  |  | x |
| Increasing Volume Response | Change in Volume from baseline to follow-up of lesion-ROI that increase. |  |  |  | x |
| Increasing SUV_hetero_ Response | Change in Mean standard deviation from baseline to follow-up of lesion-ROI that increase. |  |  |  | x |
| Decreasing SUV_max_ 1 | SUV_max_ at baseline of lesion-ROI that decrease. |  |  |  | x |
| Decreasing SUV_mean_ 1 | SUV_mean_ at baseline of lesion-ROI that decrease. |  |  |  | x |
| Decreasing SUV_total_ 1 | SUV_total_ at baseline of lesion-ROI that decrease. |  |  |  | x |
| Decreasing Volume 1 | Volume at baseline of lesion-ROI that decrease. |  |  |  | x |
| Decreasing SUV_hetero_ 1 | Mean standard deviation at baseline of lesion-ROI that decrease. |  |  |  | x |
| Decreasing Count 1 | The number of lesion-ROI at baseline that decrease. |  |  |  | x |
| Decreasing SUV_max_ 2 | SUV_max_ at follow-up of lesion-ROI that decrease. |  |  |  | x |
| Decreasing SUV_mean_ 2 | SUV_mean_ at follow-up of lesion-ROI that decrease. |  |  |  | x |
| Decreasing SUV_total_ 2 | SUV_total_ at follow-up of lesion-ROI that decrease. |  |  |  | x |
| Decreasing Volume 2 | Volume at follow-up of lesion-ROI that decrease. |  |  |  | x |
| Decreasing SUV_hetero_ 2 | Mean standard deviation of lesion-ROI that decrease. |  |  |  | x |
| Decreasing Count 2 | The number of lesion-ROI at follow-up that decrease. |  |  |  | x |
| Decreasing SUV_max_ Response | Change in SUV_max_ from baseline to follow-up of lesion-ROI that decrease. |  |  |  | x |
| Decreasing SUV_mean_ Response | Change in SUV_mean_ from baseline to follow-up of lesion-ROI that decrease. |  |  |  | x |
| Decreasing SUV_total_ Response | Change in SUV_total_ from baseline to follow-up of lesion-ROI that decrease. |  |  |  | x |
| Decreasing Volume Response | Change in Volume from baseline to follow-up of lesion-ROI that decrease. |  |  |  | x |
| Decreasing SUV_hetero_ Response | Change in Mean standard deviation from baseline to follow-up of lesion-ROI that decrease. |  |  |  | x |
| Global SUV_peak_ 1 | Highest SUV_peak_ on the baseline scan. |  |  |  |  |
| Global SUV_peak_ 2 | Highest SUV_peak_ on the follow-up scan. |  |  |  |  |
